# Wnt/β-Catenin Signaling Pathway Is a Direct Enhancer of Thyroid Transcription Factor-1 in Human Papillary Thyroid Carcinoma Cells

**DOI:** 10.1371/journal.pone.0022280

**Published:** 2011-07-21

**Authors:** Marie Gilbert-Sirieix, Joelle Makoukji, Shioko Kimura, Monique Talbot, Bernard Caillou, Charbel Massaad, Liliane Massaad-Massade

**Affiliations:** 1 UMR 8203 CNRS/Institut Gustave Roussy, Villejuif, France; 2 UMR 8194 CNRS/Université Paris Descartes, Paris, France; 3 Laboratory of Metabolism, National Cancer Institute, Bethesda, Maryland, United States of America; 4 UMR 8200 CNRS/Institut Gustave Roussy, Villejuif, France; Vanderbilt University Medical Center, United States of America

## Abstract

The Wnt/β-catenin signaling pathway is involved in the normal development of thyroid gland, but its disregulation provokes the appearance of several types of cancers, including papillary thyroid carcinomas (PTC) which are the most common thyroid tumours. The follow-up of PTC patients is based on the monitoring of serum thyroglobulin levels which is regulated by the thyroid transcription factor 1 (TTF-1): a tissue-specific transcription factor essential for the differentiation of the thyroid. We investigated whether the Wnt/β-catenin pathway might regulate TTF-1 expression in a human PTC model and examined the molecular mechanisms underlying this regulation. Immunofluorescence analysis, real time RT-PCR and Western blot studies revealed that TTF-1 as well as the major Wnt pathway components are co-expressed in TPC-1 cells and human PTC tumours. Knocking-down the Wnt/β-catenin components by siRNAs inhibited both TTF-1 transcript and protein expression, while mimicking the activation of Wnt signaling by lithium chloride induced TTF-1 gene and protein expression. Functional promoter studies and ChIP analysis showed that the Wnt/β-catenin pathway exerts its effect by means of the binding of β-catenin to TCF/LEF transcription factors on the level of an active TCF/LEF response element at [−798, −792 bp] in TTF-1 promoter. In conclusion, we demonstrated that the Wnt/β-catenin pathway is a direct and forward driver of the TTF-1 expression. The localization of TCF-4 and TTF-1 in the same area of PTC tissues might be of clinical relevance, and justifies further examination of these factors in the papillary thyroid cancers follow-up.

## Introduction

Papillary thyroid carcinoma (PTC) is the most common thyroid cancer representing about 80% of all thyroid cancers [1]. Treatment of PTC is based on total thyroidectomy and radioiodine therapy [2]. The gold-standard method in the follow-up of patients after surgery/radioiodine is based on monitoring of serum thyroglobulin (Tg) levels, which showed a higher sensitivity than cytology for the detection of cervical lymph node metastasis [3]. However, the thyroid specific transcription factor TTF-1 regulates Tg expression by binding to its promoter [4]. Therefore, TTF-1 is considered with Tg as the most important markers for follicular cells [5], [6].

The transcriptional factor TTF-1 (also known as NKX2-1, T/EBP or TITF-1) is commonly expressed in the thyroid gland, lung and central nervous system [7]. It is considered as a marker of differentiation in thyroid and lung carcinoma [8] and has been widely used to discern the primary site of thyroid and lung tumour origin in patients with metastatic disease [9], [10], [11], [12]. In the thyroid gland, TTF-1 is expressed in the follicular cells and, together with Pax8, controls the expression of Tg, thyroperoxydase (TPO), thyrotropin receptor (TSH), the sodium/iodide symporter (NIS) and calcitonin and major histocompatibility complex class I genes. Consequently, the combination of these two factors plays a role in the expression of the thyroid-specific phenotype. *TTF-1* mRNA is detected in papillary carcinomas (PTC) but not in anaplastic carcinomas; therefore TTF-1 is considered as a marker to distinguish between these two types of thyroid neoplasms [13], [14]. Concerning the prognosis, TTF-1 expression may be increased in PTC with aggressive clinical course [15].

The Wnt signaling pathway is a complex network of proteins described to be involved in the control of thyrocyte proliferation [16] and to play a pivotal role in thyroid cancer development [16]. In the absence of Wnt signal, β-catenin is targeted for degradation in the proteasome. In the presence of Wnt ligands, the Frizzled receptor is activated, leading to the repression of GSK3β and consequently to the accumulation of β-catenin and its translocation to the nucleus [17]. There, β-catenin forms a complex with the nuclear transcriptional regulator T-cell factor/lymphoid enhancer factor (TCF/LEF), to promote the expression of Wnt target genes [17]. For examples in normal thyroid tissue, Wnt-1 ligand enhances cell growth of differentiated thyroid cell and regulates thyroperoxidase gene, a critical enzyme for thyroid hormone synthesis in thyrocytes [18] and GSK3β are tightly implicated in the thyrocytes stimulation [19]. Moreover, aberrant activation of the Wnt signaling pathway may be a common denominator for the development of tumours [20] and strongly involved in thyroid tumorigenesis [21]. It was reported a dominant role of Wnt/β-catenin signaling relative to the TSH/PKA/CREB pathway in the proliferation of normal and neoplastic thyrocytes [22]. Also, aberrant β-catenin expression or localisation are often described to be associated with the more aggressive behaviour in PTCs [22]–[23] and many β-catenin target genes have been shown to play an important role in cancer progression including *c-myc*, *cyclin D1*, matrix metalloproteases, *CD44* and homeodomain-containing genes [24], [25], [26], [27], [28]. Furthermore, it has also been suggested that β-catenin may play a direct role in the dedifferentiation of the late-stage disease of PTC [16], [29].

The role of Wnt/β-catenin signaling in TTF-1 regulation remains unclear. The aim of this study is to investigate whether the Wnt/β-catenin pathway could regulate TTF-1 expression in a papillary thyroid carcinoma model and to examine the mechanism(s) involved in this regulation. We show herein that Wnt/β-catenin pathway is a direct driver of TTF-1 expression.

## Results

### The major components of the Wnt/β-catenin signaling pathway and TTF-1 are co-expressed in the TPC-1 cell line and papillary thyroid carcinomas

**Figure 1:**
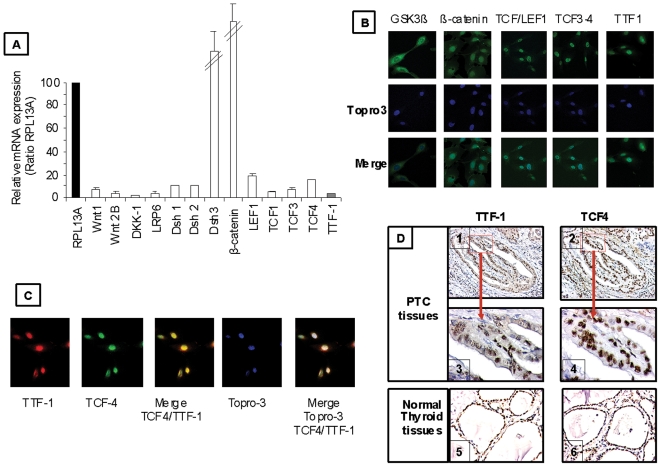
Co-expression of Wnt/β-catenin pathway components and TTF-1 in the TPC-1 cell line and PTCs. (**A**) TPC-1 cells were cultured for 24 h, total RNA was isolated, reverse transcription was performed and then cDNA was amplified by real time PCR (qRT-PCR) using primers described in Table 1. Results were compared to RPL13A expression. Data are represented as the mean of three independent experiments. (**B**) TPC-1 cells were incubated for 24 h, fixed and immunostained for GSK3-β, β-catenin, TCF/LEF1, TCF3-4 and TTF-1 with primary antibodies and Alexa 555 or 488-conjugated secondary antibodies. Cell nuclei were stained with TO-PRO3 iodide (blue) and fluorescence was assessed by fluorescence microscopy combined with confocal analysis. Representative images of three independent experiments are shown with magnification, ×60. (**C**) The expression assessment and localization of TCF-4 and TTF-1 in TPC-1 cells performed by immunocytochemical studies. TTF-1 antibody conjugated with Alexa fluor 488 (red) or TCF-4 antibody conjugated to Alexa fluor 555 (green) were used, cell nuclei were stained with TO-PRO3 iodide (blue). Fluorescence was assessed by fluorescence microscopy combined with confocal analysis (representative images of three independent experiments). (D) Immunohistochemistry experiments were carried on paraffin-embedded in papillary thyroid carcinomas (1 and 2) and in normal thyroid tissues (5 and 6) using the antibodies recognizing either TTF-1 or TCF-4, magnification ×100. Consecutive serial sections (×400) were performed for PTC tissues (3 and 4). The high power view of papillary structures corresponds to the red square on the lower magnification (×100).

We investigated by real-time PCR the relative expression in the TPC-1 cell line of TTF-1 as well as the major components of the Wnt/β-catenin pathway (Wnt1 ligand, the negative regulator Dickkopf (Dkk1), the LRP6 co-receptor, Dishevelled (Dsh1-2-3), β-catenin and the transcription factors: LEF-1, TCF-1, TCF-3, TCF-4. Most of these genes were less expressed 300-fold to 3-fold than RPL13A, except for Dsh3 (1.2-fold higher than RPL13A) and β-catenin (4.8-fold higher than RPL13A) (Figure 1A).

Immunocytochemistry (ICC) experiments were performed to determine the presence and the subcellular localization of Wnt/β-catenin signaling pathway and of TTF-1 protein in the human TPC-1 cells. As depicted in Figure 1B, GSK-3β staining was strictly cytoplasmic, while β-catenin exhibited both nuclear and cytoplasmic localization. TCF/LEF1, TCF3-4 and TTF-1 proteins were strictly nuclear. Moreover, TCF-4 and TTF-1 were co-localized in the nuclei of TPC-1 cells (Figure 1C). Interestingly, immunohistochemical studies performed on four papillary thyroid carcinomas and three normal thyroid tissues taken at distance of benign adenoma showed a TCF-4 and TTF-1 positive expression and localized in nuclei in normal tissue as well as in PTC. Immunostains performed on consecutive serial histological sections showed that both TCF-4 and TTF-1 are expressed in the same areas within tumours (Figure 1D).

Altogether, our results clearly showed that the major components of Wnt/β-catenin signaling pathway as well as TTF-1 are expressed in TPC-1 cells. Thus, we aimed to explore the potential regulation of TTF-1 expression by Wnt/β-catenin pathway.

### LRP6, Dsh, β-catenin and TCF are required for the basal expression of *TTF-1* gene

**Figure 2:**
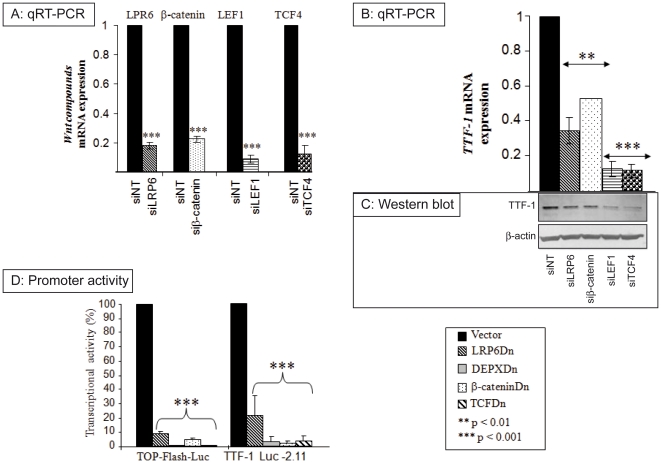
Silencing of Wnt components reduces TTF-1 levels and required for the basal expression of *TTF-1*. **A, B and C:** TPC-1 cells were transfected with 50 nM of siRNAs targeting LRP6, β-catenin, LEF1 or TCF-4, and a non-targeting siRNA sequence (siNT) as control. mRNA expression of *LRP6*, *β-catenin*, *LEF1*, *TCF4* (**A**) and *TTF-1* (**B**) were analyzed by real-time PCR and normalized to *RPL13A* mRNA, then recorded as the fold change compared to the non targeting siRNA (siNT). **A**. Significant inhibition of *LRP6*, *β-catenin*, *LEF1 and TCF4* was found for cells transfected with siRNAs targeting the corresponding genes compared with those transfected with the scrambled sequence. (**B**) Significant inhibition of *TTF-1* in cells transfected with siRNAs targeting LRP6, β-catenin, LEF1 or TCF-4. (**C**) Western blot data showing the effect of siRNAs targeting LRP6, β-catenin, LEF1 and TCF-4 on TTF-1 protein expression, β-actin was used as internal control (images from one experiment representative of three independent experiments) (**D**) TPC-1 cells were co-transfected with TOP-Flash-Luc vectors or with the TTF-1-luc-2.11 and dominant-negative plasmids (LRP6Dn, DEPXDn, β-cateninDn, TCFDn) and luciferase activity was assessed 24 h later. Results were expressed as percent of transcriptional activity as described in Material and Methods section. Columns represent three independent experiments and bar the standard deviation of the mean.

To investigate whether Wnt/β-catenin pathway controls the expression of TTF-1, we knocked-down the expression of the Wnt/β-catenin pathway major components: LRP6, β-catenin, LEF1 and TCF-4. LRP6, β-catenin, LEF1 and TCF-4 knockdown by siRNAs led to a reduction in their cognate mRNA levels by −80% −95% demonstrating the efficiency of the knockdown (Figure 2A). The silencing of Wnt pathway components potently reduced *TTF-1* mRNA (Figure 2B) and protein levels (Figure 2C).

To assess if the effect of Wnt/β-catenin is exerted on the level of TTF-1 promoter, we transfected TPC-1 cells with a luciferase reporter plasmid baring 2.11 kbp of TTF-1 promoter, with plasmids expressing dominant negative forms of either LRP6 (LRP6Dn), Dsh (DEPXDn), β-catenin (β-cateninDn) or TCF (TCFDn). The efficiency of the knockdown of Wnt/β-catenin pathway was tested on TOP-Flash-Luc construct that contains four copies of TCF/LEF response element. TOP-Flash-Luc transcriptional activity was strongly inhibited when the dominant negative forms of Wnt components were overexpressed (up to 95% inhibition) (Figure 2D , left panel). TTF-1 promoter activity was decreased by 80% when LRP6Dn was transfected, and by 95% with the DEPXDn, β-cateninDn and TCFDn (Figure 2D , right panel). These results demonstrate that the Wnt/β-catenin signaling pathway is crucial for the basal expression of the TTF-1 gene.

### The stabilization of β-catenin induces TTF-1 expression

**Figure 3:**
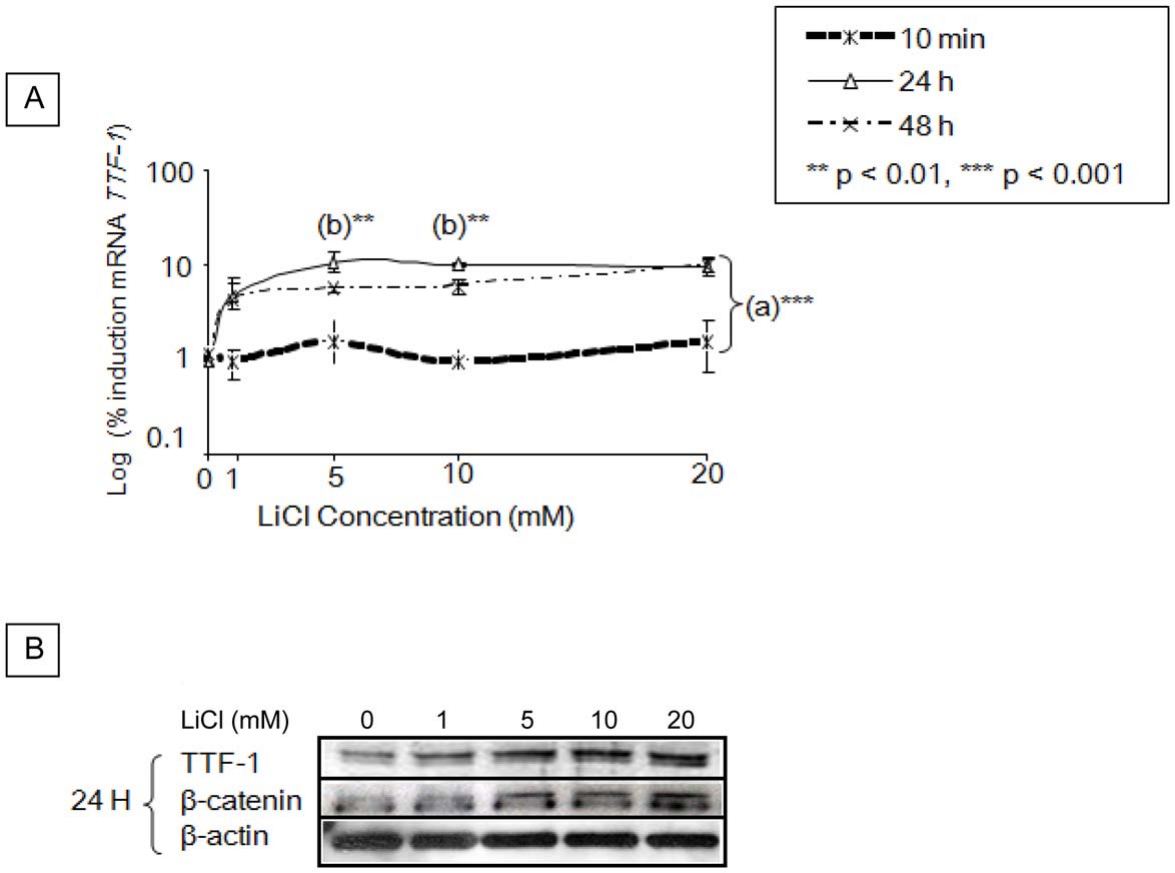
LiCl induces TTF-1 expression. (**A**) TPC-1 cells were incubated without or with 1 mM, 5 mM, 10 mM, and 20 mM of LiCl for 10 min, 24 h and 48 h. Expression of *TTF-1* mRNA was quantified by real-time PCR and normalized with *RPL13A* mRNA. Results of three independent experiments are reported as mean and standard deviation of the mean (SD). **a**: significant changes between cells cultured for 10 min and 24 h and 48 h. **b**: statistical significance between cells cultured for 24 h and 48 h. (**B**) TTF-1, β-catenin and β-actin protein levels in cells incubated for 24 h with the same LiCl concentrations; Western blot experiments were performed three times.

We then determined whether β-catenin stabilization may further increase TTF-1 gene expression. To mimic Wnt/β-catenin activation, we stabilized β-catenin by treating TPC-1 cells with a GSK-3β inhibitor: LiCl (1, 5, 10, 20 mM), for 10 min, 24 h and 48 h. We observed a 10-fold increase in the expression of TTF-1 at 24 h when cells were incubated with 5 to 20 mM LiCl (Figure 3A). This up-regulation was maintained at 48 h for all the concentrations of LiCl tested. We also observed an increase of TTF-1 mRNA expression even with 1 mM LiCl concentration. Western blot analysis at 24 h confirmed the stimulation of TTF-1 at the protein level. The enhancement of TTF-1 expression was concomitant with an increase of β-catenin protein level (Figure 3B).

### LiCl induces β-catenin recruitment on TCF/LEF binding site in the TTF-1 promoter

To pinpoint the effects of β-catenin stabilization at the level of the TTF-1 gene promoter, we adopted two strategies: (i) functional deletion of TTF-1 promoter, (ii) ChIP analysis of the recruitment of β-catenin on the level of TTF-1 promoter.

**Figure 4:**
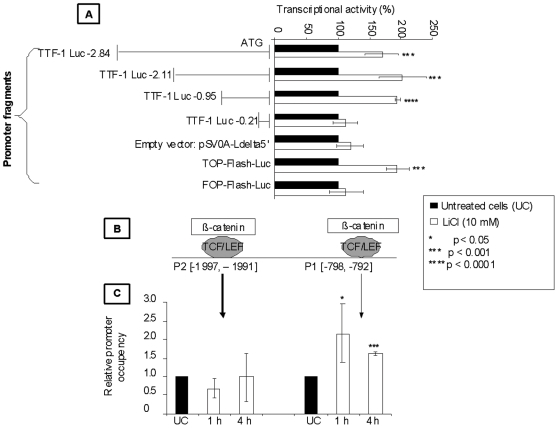
LiCl transactivates *TTF-1* promoter and induces β-catenin recruitment on TCF/LEF binding sites in *TTF-1* promoter. **A:** TPC-1 cells were transfected with pSV0AL-A(delta)5′ firefly luciferase reporter plasmids containing progressive deletions of the TTF-1 promoter fragments or with TOP-Flash-Luc and FOP-Flash-Luc. Cells were incubated in the absence or the presence of 10 mM LiCl for 24 h then luciferase activity was assessed. Results from three independent experiments displayed mean ± SD. *p* value: significant change compared with cells transfected under normoxia (Mann-Whitney *U* test). (**B**) TCF/LEF consensus sequences found in the TTF-1 promoter were localised by using the Genomatix software. (**C**) TPC-1 cells were incubated with or without 10 mM LiCl. ChIP was performed 1 h and 4 h later. Real time RT-PCR was achieved using β-catenin primers to detect the active promoter site between the two selected sites. *p*<0.05 and 0.01 and 0.001 significant change compared to control (ANOVA; Bonferroni's test). Columns, mean of at least three independent experiments; bars, SD.

First, the effect of LiCl on the regulation of TTF-1 transcription was investigated by transfecting TPC-1 cells with a reporter plasmid coupled to different fragments of the TTF-1 promoter (Figure 4A). TOP-Flash-Luc and FOP-Flash-Luc (the TCF/LEF sites were inactivated by point-mutations) were used as positive and negative controls, respectively. Cells transfected with the TTF-1 promoter fragments [TTF-1 Luc-2.84, TTF-1 Luc-2.11, TTF-1 Luc-0.95] as well as TOP-Flash-Luc displayed approximately a 2-fold increase in luciferase reporter activity after incubation with LiCl as compared to untreated cells. The luciferase activities were not enhanced by LiCl in the cells transfected with the smaller promoter fragment [TTF-1 Luc-0.21], the empty vector (pSV0A-Ldelta5′) or the FOP-Flash-Luc. These results suggest the presence of a TCF/LEF responsive element between 0.95 and 0.21 kbp upstream of the transcription start site which could be responsible for the TTF-1 up-regulation by LiCl.

*In silico* analysis of the TTF-1 promoter sequence up to −2840 bp from the ATG (available in the human genome database Ensembl) revealed by Genomatix software the presence of two putative binding sites for TCF/LEF (Figure 4B). The first one corresponds to the P1 location [−798, −792 bp] while the second one is located between [−1997, −1991 bp] and here referred to as P2. To verify the effective recruitment of β-catenin on the putative TCF/LEF binding sites, we conducted three ChIP assays on TPC-1 cell extracts treated with LiCl (10 mM for 1H and 4H). A 2-fold stimulation of β-catenin recruitment was observed after 1H and 4H of LiCl but only for the P1 site (Figure 4C). The P2 site [−1997, −1991 bp] did not further recruit β-catenin. These results indicate that the [−798, −792] region is necessary for the TTF-1 transcriptional activation by TCF/β-catenin complex.

## Discussion

TTF-1 plays a very important role in the development, cell growth and differentiation process of thyroid. TTF-1 is expressed in thyrocytes where it regulates the activity of specific genes related to the thyroid function. In neoplastic tissues, TTF-1 is used as a marker of differentiation in thyroid and lung carcinomas [8] and has been widely used to discern the primary site of thyroid and lung tumour origin in patients with metastatic disease [9], [10], [11], [12]. Importantly, TTF-1 is expressed at lower levels in malignant thyroid as compared with normal thyroid tissue and is also used as marker of thyroid differentiation [13]. It has been suggested that normal expression of Wnt/β-catenin signaling pathway have a functional relevance in the control of thyrocyte proliferation [22] and its dysregulation represents a key step in neoplastic thyrocyte proliferation [21], [29], [30]. The study of the cross-talk between Wnt/β-catenin pathway and TTF-1 is of great interest to understand the normal development of thyroid and its transformation into carcinoma. Therefore, we investigated whether the Wnt/β-catenin pathway regulates TTF-1 expression in a human PTC model (TPC-1) and examined the inherent mechanism(s).

First, we verified the TTF-1 expression in the human TPC-1 cell line and in human papillary thyroid carcinoma by qRT-PCR and immunofluorescence. In both cases, we showed the nuclear localization of the TTF-1 protein. We started with these experiments because several authors failed to find a TTF-1 expression in TPC-1 cells [31], [32], [33]. While, in contrast, others reported its presence in the same cell line [34], [35]. The discrepancy between these reports might be explained by the emergence of novel strains of cell lines due to self-selection or mutations, as suggested by Pilli *et al.*, [36] or to technical problems due to the sensitivity of the techniques used for the detection.

The molecular partners of the Wnt/β-catenin pathway analysed in our study are expressed and their proteins localized in TPC-1 cells. We then investigated whether the Wnt/β-catenin pathway regulates TTF-1 and two strategies were adopted.

The first one consists of knocking-down the molecular compounds of Wnt/β-catenin upstream (*LRP6*, *Dsh*) or downstream (*TCF-4*) of the *β-catenin*. We found a downregulation of TTF-1 mRNA and protein. The loss-of-function experiments revealed a strong inhibition of the transcriptional TTF-1 activity with dominant negative LRP6, Dsh, β-catenin and TCF expression vectors. Taking together, these findings confirm that the molecular partners of Wnt/β-catenin are responsible for the TTF-1 regulation by post-transcriptional effects influencing the TTF-1 mRNA and protein expression and that they are required for the basal expression of the TTF-1 promoter.

The second strategy consists of mimicking the effects of Wnt signaling activation through GSK-3β inhibition by LiCl [37], [38]. An up-regulation of TTF-1 mRNA and protein was observed. Moreover, promoter studies indicated the presence of putative binding sites for TCF/LEF between 0.95 and 0.21 kbp upstream the ATG responsible of the LiCl effect. Indeed, ChIP experiments demonstrated that the TCF/LEF responsive element [−798; −792] is responsible for LiCl-induced β-catenin recruitment on TCF/LEF binding site in the TTF-1 promoter.

In conclusion, the present study has a double impact. First, from a fundamental point of view, this work demonstrates for the first time that Wnt/β-catenin regulates TTF-1 in papillary thyroid cancer cells through β-catenin-binding to a TCF/LEF-responsive element present in TTF-1 promoter. This will influence TTF-1 mRNA and protein expression. We suggest that Wnt/β-catenin pathway contributes to the fine-tuning of TTF-1 expression and could have different biological consequences according to the cellular context. We speculate that a normal Wnt/β-catenin expression maintains the basal expression of TTF-1 expression and a differentiated cell state, whereas activation of Wnt/β-catenin signaling would have an effect on tumour progression (occurs consequently to mutations within the molecular Wnt signaling partners leading to a continuous activation of β-catenin and thus of TTF-1) or tumour regression (through inactivation of GSK-3β by chemical molecules such as LiCl leading to moderate TTF-1 activation).

From the medical point of view, inactivation of GSK-3β by LiCl alone [39] or in combination with HDACi [40] was found to be sufficient to inhibit growth in medullary thyroid cancer cells. We hope that the administration of LiCl or other GSK-3β inhibitors or Wnt modulators will stimulate the expression of TTF-1 and, as a consequence, promotes thyroid cells differentiation in pathologies where TTF-1 expression is weak or lacking. This is of great interest for thyroid or lung cancers where TTF-1 expression is crucial to maintain cell differentiation. Finally, the localization of TCF-4 and TTF-1 in the same area of PTC tissues might also be of clinical interest, and justifies further examination of these factors in the follow-up of papillary thyroid cancer. Knowing that, TTF-1 is a tissue specific transcription factor, the relevance of this observation could also be investigated in tissues where TTF-1 is also expressed such as the lung and the diencephalon.

## Materials and Methods

### Ethics Statement

The study was approved by the Ethical Committee of the Institut Gustave Roussy. Patients gave written informed consent. No industry gave support for this study.

### Cell line

The human TPC-1 cell line was kindly provided by Dr. C. Dupuy (FRE2939 CNRS, IGR, France). The cell line was grown in Dulbecco's Modified Eagle Medium (DMEM) (Invitrogen, Cergy-Pontoise, France) supplemented with 10% fetal bovine serum (FBS), 100 units/ml penicillin and 100 µg/ml streptomycin (Invitrogen). Cells were incubated at 37°C in a humidified atmosphere containing 5% CO_2_.

### Immunocytochemistry

TPC-1 cells were plated in 6-well plates with coverslide at a density of 2×10^4^ cells per well. The protocol used in this study was as previously described [41]. The following primary antibodies were used: rabbit polyclonal anti-β-catenin (1∶100, Sigma, France), rabbit polyclonal anti-GSK-3β (1∶100, Millipore, France), mouse monoclonal anti- TCF/LEF-1 (1∶100, Millipore, France), mouse monoclonal anti-TCF3-4 (1∶100, Abcam, USA), mouse monoclonal TCF-4 (1∶100, Millipore, France) and rabbit polyclonal anti-TTF-1 (1∶100, Upstate, USA). Cells were labeled with conjugated antibodies (Alexa fluor 555 or 488, Invitrogen, France) for 1 h in the dark in a humidified chamber and nuclei of cells were detected with TO-PRO3 Iodide (Invitrogen, France). After final washes, the coverslips were mounted on slide using Fluoromount-G (SouthernBiotech, USA) and cells were analyzed with a Zeiss LSM 510 confocal microscope.

### Immunohistochemistry

Human thyroid tissues embedded in paraffin blocks were obtained from archive files of Institut Gustave-Roussy, pathology department. They included four primary papillary carcinoma and three apparently normal thyroid tissues taken at distance of benign adenoma. An immunohistochemical study was done on freshly cut 4-µm serial sections with anti-TTF1 (Upstate) and anti-TCF4 (Millipore). Immune complexes were visualized using HRP anti-Rabbit (DAKO- Carpinteria, ref: K 4011) and DAB chromogen solution (DAKO, ref. K 3468). Nuclei were lightly counterstained with hematoxylin and sections were mounted with Histomount (National Diagnostics).

### Plasmids, siRNAs and Chemicals

Firefly luciferase reporter plasmids containing various regions of the TTF-1 promoter (TTF-1-luc-2.84, TTF-1-luc-2.11, TTF-1-luc-0.95, TTF-1-luc-0.21) and the control empty vector (pSV0AL-A(delta)5′) were used as previously described [42]. TOP-Flash-luc, FOP-Flash-luc plasmids and PCS2+ vectors carrying cDNA fragments encoding dominant-negative form of LRP6 (LRP6Dn) [43], of Dishevelled (DEPXDn), of β-catenin (β-cateninDn) and of TCF (TCFDn) were kindly provided by Dr C. Fonte (UMR788, Inserm le Kremlin-Bicêtre, France). LRP6, β-catenin (CTNNB1), LEF1 and TCF-4 (TCF7L2) SMARTpool ON-TARGET-plus siRNAs and non-targeting siRNA (NT) were purchased from Dharmacon (Lafayette, CO). Each gene was targeted with four different sequences of siRNA directed against four different regions of the cognate mRNA. Lithium chloride (LiCl) was purchased from Sigma-Aldrich (France).

### Transient transfections

To knockdown some of the Wnt/β-catenin pathway molecules (LRP6, β-catenin, LEF1 and TCF-4) transient transfections were carried out using Lipofectamine 2000 transfection reagent (Invitrogen). Briefly, 24 h before transfection, 3×10^5^ TPC-1 cells were seeded in 6-well plates. Transfections were performed in serum-free OPTI-MEM using a 50 nM siRNAs and 6 µl Lipofectamine. Cells were incubated with transfectants for 48 h.

For promoter studies, transient transfection of plasmids was carried out using the jetPEI™ reagent (PolyPlus Transfection, France) according to the manufacturer's instructions. We used 3 µg of TTF-1-luc plasmids or 0.5 µg of TOP-Flash-luc or FOP-Flash-luc. Twenty four hours after transfection, cells were exposed to LiCl (10 mM) for 24 hours. After harvesting, cell extracts were assayed for luciferase activity (Luciferase Assay System, Promega, Charbonnieres, France) and luminescence was measured in a Lumat LB 9507 luminometer (Berthold France SA, Thoiry, France). The luciferase activity was normalized to the protein levels (assessed by Micro BCA™ Protein Assay Kit, Pierce, Brebieres, France).

The same protocol was used for transient transfections with dominant-negative expression vectors. TPC-1 cells were cotransfected with 3 µg of TTF-1-luc-2.11 plasmid and 1 µg of dominant-negative expression vectors. Results were expressed as percent of transcriptional activity: [luciferase activity elicited by each sample×100]/luciferase activity elicited by the TOP-Flash-Luc plasmid alone or TTF-1 luc-2.11 alone].

### Lithium treatment

To determine the effect of LiCl (an inhibitor of GSK-3β) on TTF-1 expression, kinetic studies were performed on TPC-1. Cells were plated in 6-well plates at 3×10^4^cells/well and incubated with different LiCl concentrations (0, 5, 10, 20 mM) for 10 min, 24 h and 48 h. qRT-PCR and Western Blot were performed after harvesting the cells.

### Real time PCR (qRT-PCR)

Total RNA was extracted from TPC-1 cells using RNeasy mini-kit (Qiagen, Courtaboeuf, France). First-strand cDNA was generated with M-MLV RT buffer pack (Promega France, Charbonnières-les-Bains, France).

Primers used for Real-time PCR (qRT-PCR) are described in Table 1. qRT-PCR was carried out with the ABI PRISM 7000 Taqman (Applied Biosystems, Perkin-Elmer) using SYBR GreenER qPCR Supermix for ABI PRISM (Invitrogen) according to manufacturer's instructions. All PCRs were performed in duplicate. Gene regulation was determined by the 2^−ΔΔCt^ method [44] and normalized or compared to RPL13A (ribosomal protein) levels [41]. For mRNA relative expression of TTF-1 and of Wnt/β-catenin components, results are given as relative fold compared to RPL13A. For knockdown experiments results are given as relative fold compared to the non targeting siRNA (siNT). For LiCl experiments results are given as relative fold compared to untreated cells.

**Table 1:** Primers sequences used for real time-RT-PCR analysis.

Gene name	Primer sequence	Position	Length (bp)	Tm (°C)
**WNT1**	F:CTCTCTTCTTCCCCTTTGTC	1685–2029	345	59°C
	R:AACTCGTGGCTCTGTATCC			
**WNT2B**	F:GATGGGACAAAGATGAATGG	1647–1928	282	55°C
	R:GGCAGCAAGTAAGCAAAAG			
**DKK1**	F:GAGTCCTTCTTCTGAGATGATGG	142–404	263	61°C
	R:GGTACGGCTGGTAGTTGTC			
**LRP5**	F:GTGTGTGACAGCGACTACAG	4714–4890	177	60°C
	R:CGGGAAGAGATGGAAGTAG			
**LRP6**	F:CAGGGTGGAATGAATGTGC	2740–2963	224	58°C
	R:GTGGATGGGAAGGATGATG			
**Dsh1**	F:TCAACGGAAGGGTGGTATC	1373–1665	293	62°C
	R:GGCTGCTGGACACATTAGG			
**Dsh2**	F:AACCCCTGCGAGTTCTTC	337–525	189	60°C
	R:ATCCCCACCAGACACAAG			
**Dsh3**	F:CCTCCATCACCAGTTCCAC	1234–1420	187	59°C
	R:AGCCAGTCCACCACATCTG			
**β-catenin**	F:AAAATGGCAGTGCGTTTAG	1076–1175	99	56°C
	R:TTTGAAGGCAGTCTGTCGTA			
**LEF1**	F:CCTGGTCCCCACACAACTG	1405–1535	131	58°C
	R:GGCTCCTGCTCCTTTCTCTG			
**TCF1**	F:GACATCAGCCAGAAGCAAG	725–866	142	58°C
	R:CACCAGAACCTAGCATCAAG			
**TCF3**	F:GTACCCCTTCCTGATGATCC	437–580	144	60°C
	R:GACCTCGTGTCCTTGACTG			
**TCF4**	R:CCACATCATACGCTACACAC	913–1119	207	55°C
	F:GACCTTTGCTCTCATTTCC			
**TTF-1**	F:CCAGAACCACCGCTACAA	627–805	179	62°C
	R:GTTTGCCGTCTTTCACCA			

F = forward primer, R = reverse primer.

### Protein extraction and Western blot analysis

Total protein extracts were obtained using M-PER reagent (Pierce) with protease inhibitors cocktail (Roche, Neuilly, France). Proteins were titrated by the BCA method using the BCA protein assay (Thermo Scientific/Pierce, Courtaboeuf, France). Cell extracts were separated on 10% SDS-PAGE gel. Proteins were transferred using the iBlot™ Dry Blotting System (Invitrogen) on pre-cut nitrocellulose membranes, then blocked with 0.2% casein (I-Block reagent; Tropix, Bedford, MA) in PBS with 0.1% Tween-20. The membranes were incubated overnight at 4°C with the primary antibody specific for TTF-1 (Upstate Cell Signaling, 1∶300; Danvers, MA USA), β-catenin (rabbit polyclonal, 1∶2000, Sigma-Aldrich Chimie S.a.r.l. Lyon, France) or β-actin (rabbit polyclonal, 1∶1000, Sigma-Aldrich) used as internal control. Blots were washed and incubated with anti-rabbit antibody conjugated to alkaline phosphatase (1∶20,000, Tropix) for 1H at room temperature, and subsequently washed and revealed using CDP Star chemoluminescence reagent (Perkin Elmer).

### Real-time chromatin immunoprecipitation (ChIP) assay

TPC-1 cells were plated in 10 cm plates (3×10^6^cells/plate) and treated or not with LiCl for 1 h and 4 h then fixed with 1% formaldehyde added to the medium for 10 min, scraped, and collected by centrifugation. Cells were resuspended in 300 µl of lysis buffer (5 mM piperazine-N,N′-bis(2-ethanosulfonic acid) (PIPES pH 8.0), 85 mM KCl, 0.5% NP-40) with a cocktail of protease inhibitors (Roche). Cells were pelleted by centrifugation and suspended in 300 µl of 1% SDS, 10 mM EDTA, and 50 mM Tris-HCl (pH 8.0) containing protease inhibitors. After incubation on ice for 10 min, they were sonicated six times for 30 seconds using Bioruptor (Diagenode). Lysates were then cleared by centrifugation and the concentration of DNA was determined. Equal amounts of DNA were diluted ten times in dilution-buffer (0.01% SDS, 1% Triton X-100, 1.2 mM EDTA, 16.7 mM Tris-HCl (pH 8.1), 167 mM NaCl). The chromatin solution was precleared for one hour at 4°C on Protein G-Agarose/Salmon Sperm DNA beads from Upstate. After brief centrifugation and removal of the beads, DNA was incubated overnight at 4°C on a rotating wheel with 1 µg of either anti-β-catenin, 2206, Sigma) or non-relevant antibody (anti-nucleolin C23, SC-13057, Santa Cruz biotechnology). Immune complexes were collected on Protein G-Agarose/Salmon Sperm DNA beads from Upstate. Beads were washed sequentially in TSE (0.1% SDS, 1% Triton X-100, 2 mM EDTA, 20 mM Tris-HCl (pH 8.1), with 150 mM NaCl, TSE with 500 mM NaCl, buffer A (0.25 M LiCl, 1% NP-40, 1% deoxycholate, 1 mM EDTA, 10 mM Tris-HCl [pH 8.1]), and twice with Tris-EDTA and then eluted with 200 µl 1% SDS and 0.1 M NaHCO3. Cross-links were reversed by heating at 65°C for 4 h after adding NaCl to 200 mM final concentration. After treatment with Proteinase K (50 µg/ml) for 1 h at 37°C, DNA was purified using Geneclean Turbo kit (Q-Biogene). Next, real-time PCR analysis of inputs and immunoprecipitated DNA samples were performed using the following primers: P1F: CCCTCTTCCTCCTAAGCAGTTTC, P1R: TTAAAGTGGTGGCGTAAATGGC. P2F CCCAACTCTTCCTTTTACTCAG, P2R: GTCACCCAGCTTCTTTCTCTC. Data are compared to input and normalized to anti-nucleolin C23 and expressed as 2^−ΔΔCt^ to determine the assay site IP-fold enrichment. The results are given as relative fold in site occupancy compared with cells cultured without LiCl.

### Statistical analysis

Data are represented as mean ± SD. Mann-Whitney *U* test was used to compare the mean of *Wnt* compounds mRNA relative levels after transfection with siRNAs and the mean of transcriptional activity after transfection with plasmids. For all other studies, means of treatment groups were compared with one-way ANOVA. When the ANOVA showed that there were significant differences between the groups, Dunnett's test or Bonferroni's test was used to identify the sources of these differences. *p*≤0.05 was considered statistically significant.
